# Enhanced Atomic Desorption of 209 and 210 Francium from Organic Coating

**DOI:** 10.1038/s41598-017-04397-y

**Published:** 2017-06-23

**Authors:** Steinn Agustsson, Giovanni Bianchi, Roberto Calabrese, Lorenzo Corradi, Antonio Dainelli, Alen Khanbekyan, Carmela Marinelli, Emilio Mariotti, Luca Marmugi, Leonardo Ricci, Leonardo Stiaccini, Luca Tomassetti, Andrea Vanella

**Affiliations:** 1 DSFTA, University of Siena and INFN - PI, via Roma 56, 53100 Siena, Italy; 2 Department of Physics and Earth Sciences, University of Ferrara and INFN, via Saragat 1, 44122 Ferrara, Italy; 3 INFN - Laboratori Nazionali di Legnaro, viale dell’Universit `a 2, 35020 Legnaro (PD), Italy; 4 Department of Physics and Astronomy, University College London, Gower Street, London, WC1E 6BT UK; 5 Physics Department, University of Trento, via Sommarive 14, 38123 Trento, Italy; 6 Department of Mathematics and Computer Science, University of Ferrara and INFN, via Saragat 1, 44122 Ferrara, Italy

## Abstract

Controlled atomic desorption from organic Poly-DiMethylSiloxane coating is demonstrated for improving the loading efficiency of ^209,210^Fr magneto-optical traps. A three times increase in the cold atoms population is obtained with contact-less pulsed light-induced desorption, applied to different isotopes, either bosonic or fermionic, of Francium. A six times increase of ^210^Fr population is obtained with a desorption mechanism based on direct charge transfer from a triboelectric probe to the adatom-organic coating complex. Our findings provide new insight on the microscopic mechanisms of atomic desorption from organic coatings. Our results, obtained at room temperature so as to preserve ideal vacuum conditions, represent concrete alternatives, independent from the atomic species in use, for high-efficiency laser cooling in critical conditions.

## Introduction

Organic coatings on the inner surface of vapour cells are powerful tools whenever preservation of the atomic spin orientation or alignment and/or reduction of atomic losses after collisions against the chamber are required^1^. Coated surfaces have already been widely investigated and the quasi-elastic nature of the atom-coated wall interaction has been highlighted in different contexts^2, 3^. However, there are still many details to be explored. As an example, the preparation of a coated cell usually implies specific and empirical approaches rather than a standard protocol with full control of the involved physical and chemical processes^4, 5^. In particular, atomic desorption from organic coatings still presents not completely understood aspects and it has been tested only with a limited number of stable species^6^.

In this work, we present evidence of two different desorption processes in coated cell devoted to laser cooling of radioactive Francium isotopes (^209,210^Fr). We have observed relevant desorption of Fr atoms from the Poly-DiMethylSiloxane (PDMS) coating of the cell via: (1) Contact-less Light-Induced Atomic Desorption (LIAD) produced by a broadband pulsed illumination, and (2) Contact desorption triggered by charge transfer from a triboelectrically charged dielectric probe. The desorbed atoms produce a significant increase in a Magneto-Optical Trap (MOT) population. We show that desorption of the adsorbed atoms allows for increased MOT loading rate and trapping efficiency even in critical conditions such as those of laser cooling of radioactive atoms.

These effects are reported here for the first time in the case of radioactive isotopes desorbed from an organic coating, and at room temperature. This allows to maintain the ideal conditions to carry out cold atoms experiments^7^. Comparison of the results obtained with ^209^Fr and ^210^Fr indicates that the observed processes do not depend on the characteristics of the alkali atom, its half-life, or its mass number. In addition, our observations shed new light on the details of the atomic desorption from organic coatings. Together with recent observations from metal surfaces with stable^7, 8^ and radioactive^9^ elements, our results further extend the range of applications where atomic desorption has or will have a relevant impact.

## Results

Details on the experimental setup are provided in Methods. Fr is produced via a nuclear fusion-evaporation reaction and it is extracted in its ionic form, ^*A*^Fr^+^, where *A* = 209, 210 in the present case. Fr^+^ are routed to the experimental chamber, a Pyrex cell coated with PDMS, continuously evacuated down to <10^−9^ mbar. Within the cell, in order to obtain atoms in the neutral charge state, Fr^+^ ions are implanted in a thin foil of Y (“neutraliser”). During normal operation, the foil can be resistively heated up to 1000 K. Upon implantation, an extra electron is acquired by the selected isotope (neutralisation), so that neutral Fr atoms are released in the free volume of the chamber. Here, they are eventually captured by laser beams, cooled down and trapped in a MOT^10^.

**Figure 1:**
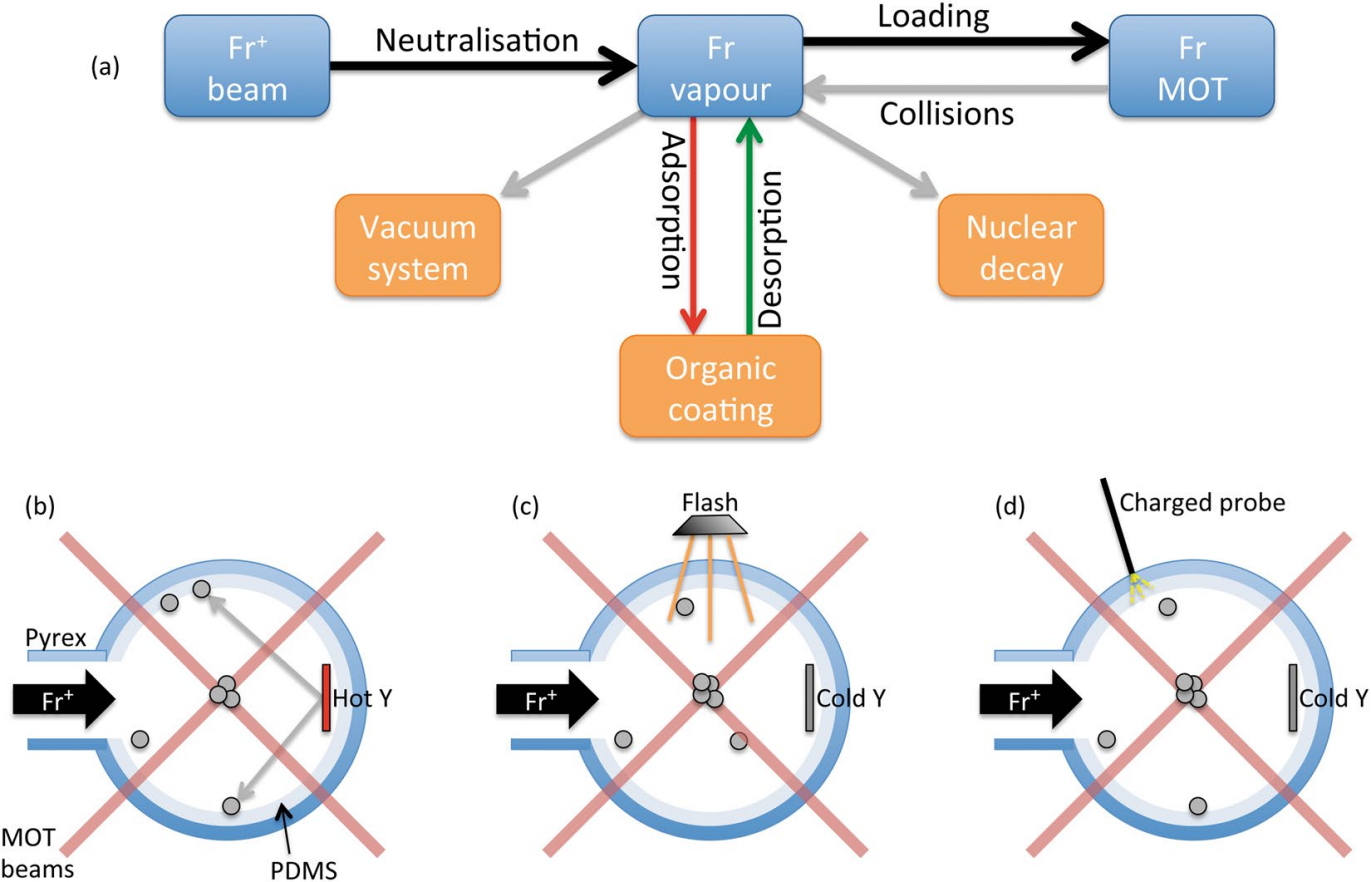
Atomic desorption processes for laser cooling of Francium isotopes. (**a**) Interplay between the source of Fr, the vapour phase, the MOT population and the main sources of loss of atomic population. Adsorption can be effectively contrasted by controlled desorption mechanisms, as discussed in the main text. (**b**) Normal operation loading of the Fr MOT. (**c**) Loading of the Fr MOT with pulsed photodesorption of atoms embedded in the PDMS coating. (**d**) Loading of the Fr MOT with charge transfer-induced atomic desorption. Sketches are not to scale and are shown for illustrative purposes only.

A complex interplay is produced among the Fr ^+^ beam, the vapour phase and the MOT, as sketched in Fig. 1(a). In addition, continuous losses further reduce the atomic population in the magneto-optical trap^9^. In particular, Fr atoms are lost because of: (i) Nuclear decay (${t}_{h.l.}^{209}=50.0\,{\rm{s}},{t}_{h.l.}^{210}=199\,{\rm{s}}$); (ii) Pumping by the vacuum system; (iii) Adsorption in the PDMS coating. While the first two causes are unavoidable, we address directly the last point: as depicted in Fig. 1(a), the desorption mechanisms described here allow for direct compensation of adsorption losses, thus producing significant increase of the MOT population. This is paramount in critical conditions such as those imposed by short-living radioactive isotopes.

Figures 1(b–d) summarise the different configurations explored in this work. Figure 1(b) represents normal operation, where Fr atoms are released from the hot Y (T = 1000 K) in the vapour phase. Atom-wall collisions lead to permanent adsorption of the atom in the Pyrex substrate (chemisorption) or to atomic depolarisation, which is detrimental for laser cooling. PDMS, as well as other organic coatings, reduces the likelihood of these phenomena^11, 12^. Nevertheless, some atoms remain trapped at the surface of the coating (physisorption), thus reducing the vapour density available for the MOT. This problem can be overcome by suitable mechanisms of desorption, which release the adatoms back in the vapour where they are captured by the cooling lasers: by using a pulse of light illuminating the coating surface, via the so-called LIAD effect, observed here from PDMS with ^209,210^Fr as sketched in Fig. 1(c); alternatively, via local charge transfer from a charged dielectric probe as depicted in Fig. 1(d). Both these approaches allow operation with room temperature Y, with immediate advantages in terms of power consumption, setup degradation and vacuum conditions.

### Light Induced Atomic Desorption of ^210^Fr

**Figure 2:**
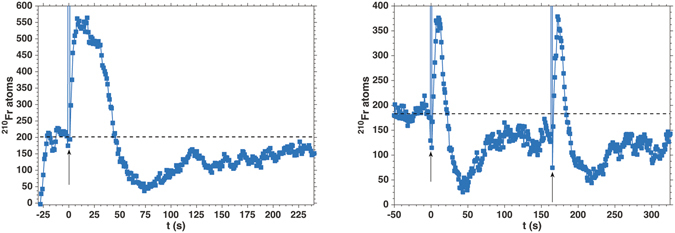
Pulsed LIAD of ^210^Fr from PDMS. **Left**: ^210^Fr MOT population time evolution after a broadband light pulse at t = 0 s. **Right**: Subsequent desorption events produced by repeated flashes exhibit similar characteristics. Arrows mark the timestamp of the flash. Horizontal dashed lines indicate the equilibrium population.

A typical result for LIAD (see also Fig. 1(c)) is shown in Fig. 2 (left) in the case of ^210^Fr, where the fluorescence produced by the MOT is used to continuously monitor MOT population changes^13^. The MOT was loaded with the neutraliser at room temperature. This operation ensures ideal vacuum conditions, at the expenses of availability of Fr atoms diffused out from the Y foil. In equilibrium conditions, the initial number of atoms is about 200, as indicated by the horizontal dashed lines in Fig. 2.

The LIAD effect is triggered by a broadband, visible flash pulse of 2 ms duration and a maximum fluence of the order of 10^−1^J/cm^2^. The light spectrum corresponds to that of a blackbody source around 5500 K. The flash light source is placed directly above the metal/glass adapter connecting the MOT cell to the ion beam line, about 10 cm from the center of the MOT. Despite a narrowband interference filter (centred at 718 ± 3 nm), the flash light produces fast saturation of the CCD. Consequently, a peak appears in the experimental traces, which is used as a marker of the light-triggered desorption (vertical arrows in Fig. 2).

The energy carried by the desorbing photons breaks the bonds of the adatoms, thus generating a net flux of Francium atoms towards the vapour phase that are rapidly cooled and captured in the MOT. Presence of Fr atoms in the coating has been confirmed by monitoring the secondary radiation, mainly *α* and *β*, emitted by Fr decay with a Geiger counter. In particular, larger counts are detected in the neutraliser region, where the ion beam is focused, and in the region of application of the flash pulse. We note that after a few tens of minutes without the incoming ion beam counts become comparable to the background, due to the short half-lives of the produced Francium isotopes. Therefore, large accumulation of Fr in coating over long timescales is excluded.

Upon light-induced desorption, ^210^Fr population increases up to 570, with 190% relative increase. Remarkably, the loading rate (60 atoms s ^−1^) is two times faster than the standard loading of ^210^Fr from vapour (27 atoms s^−1^). This is caused by the rapidly produced burst of Fr atoms released in the vapour phase by LIAD and the linear dependence of the loading rate on the vapour density. By assuming a uniform vapour density right after the photodesorption, one can estimate the number of atoms in vapour phase after LIAD from a room-temperature Maxwell-Boltzmann velocity distribution^12^, after thermalisation in the coating. In this case, we obtain a maximum number of atoms ${N}_{LIAD}^{(210)}\approx {10}^{5}$ atoms.

Before approaching again the initial level in about 200 s, the MOT population decreases down to 50 atoms. This behaviour is due to the large efficiency of the desorbing light, which depletes the coating surface of Fr atoms, and to the large area of the illuminated coating. This process increases the adsorption rate from the vapour phase to the coating, thus momentarily reducing the atomic reservoir available for laser cooling. The initial equilibrium of the whole system (see also Fig. 1(a)), and consequently the initial MOT population, are then restored. However, the return to equilibrium occurs on a time scale that is two orders of magnitude slower than the increase in the trapped ^210^Fr population upon illumination.

Such behaviour is confirmed by sequences of subsequent flash pulses, as reported in Fig. 2 (right). In Fig. 2 (right), the two peaks after the flashes correspond to the same variation in the number of trapped atoms, demonstrating the reproducibility of the loading method and the full reversibility of the desorption.

Similarly to a different experimental context^9^, it is here worth remarking that the Francium isotopes are trapped with the Yttrium neutraliser at room temperature. This is a remarkable result given the low diffusion efficiency^14^. Previous works have indeed shown that the Fr vapour density is typically maximised with a hot neutraliser:^15, 16^ higher temperature regimes favour the diffusion of atoms outside the Y surface after implantation at some tens of nanometers, as in the case of an ion beam of 3 keV like the one used in the present work. As a consequence of the exposure to a stable K vapour during passivation, a thin layer of K is formed at the Y foil surface. The presence of K in the Y foil was confirmed with independent vapour spectroscopy measurements upon heating of the neutraliser. K atoms in this layer can effectively neutralise Francium ions by transferring an extra electron, by one-to-one charge transfer^17–19^.

Nevertheless, at the current stage of investigation, we cannot rule out contributions from other mechanisms, such as sputtering, which would be however enhanced at high temperatures and with local melting^20^, and neutralisation at the Y surface. The latter is - in principle - not efficient because of the low back-scattering probability of massive Fr from the lighter Y^21^ and the relatively deep implantation depth of 3 keV Fr^+^, as estimated by means of Transport in Ions of Matter (TRIM) simulations. However, it cannot be completely neglected because a small tail of the Fr^+^ distribution is likely implanted at surface sites in the neutraliser foil. Anyway, the *direct* trapping of atoms emitted from the Y foil would be poorly efficient as the MOT capture velocity is only about 30 m/s, one order of magnitude smaller than typical velocity of atoms exiting the neutraliser.

### Light Induced Atomic Desorption of ^209^Fr

**Figure 3:**
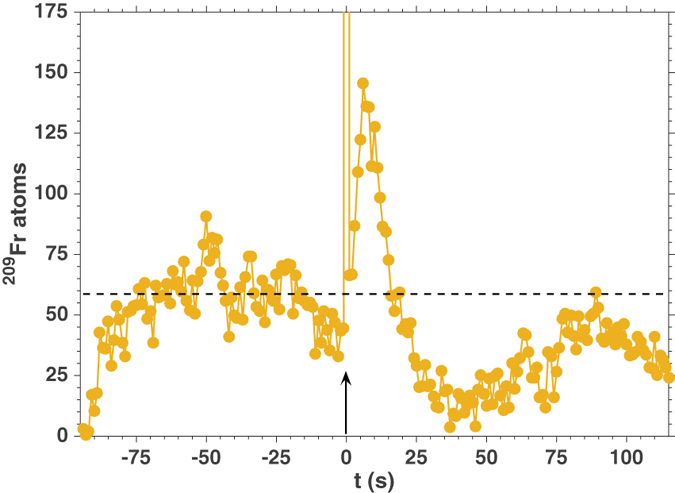
Pulsed LIAD of ^209^Fr from PDMS. ^209^Fr MOT population time evolution after a broadband light pulse at t = 0 s. The vertical arrow marks the beginning of LIAD. The horizontal dashed line indicates the equilibrium MOT population.

Both LIAD (Fig. 1(c)) from the organic coating and the possibility of “cold” Y neutralisation are independent from the fermionic or bosonic nature of the isotope, or its half-life: in Fig. 3, we present fast MOT loading from pulsed LIAD in the case of ^209^Fr.

The number of trapped atoms is smaller as a consequence of smaller energy-integrated cross-sections for the fusion-evaporation production of this isotope^22^. Nevertheless, the dynamics is similar to that observed in the case of ^210^Fr. Accordingly, a relative increase of about 200% is observed, thus with the same yield as ^210^Fr. This confirms the general character of LIAD. In particular, LIAD depends on the coating’s properties rather than on those of the alkali species, as shown also by previous experiments on stable Rubidium and Caesium in sealed cells^23^. We note that, in this case, ${N}_{LIAD}^{(209)}\approx 3\times {10}^{4}$ atoms.

### Desorption via Charge Transfer Of ^210^Fr

**Figure 4:**
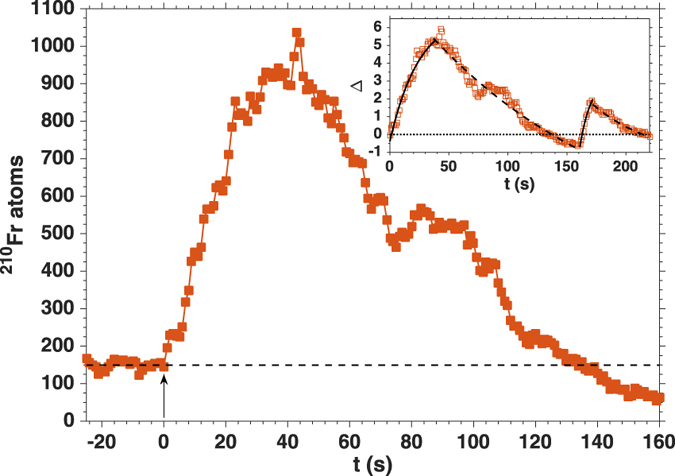
Charge transfer-induced desorption of ^210^Fr from PDMS. ^210^Fr MOT population after local charge transfer from a dielectric probe at t = 0. The horizontal dashed line indicates the MOT equilibrium population. **Inset:** Relative variation of the ^210^Fr MOT population Δ (Equation 1) in the same experiment, with a second application at t = 161 s of the charge transfer from the dielectric probe. Continuous and dashed curves correspond to the best exponential fits of experimental data.

Among the not yet explained details of LIAD from organic coatings, the desorption microscopic mechanisms are probably the most important ones^6^. We have observed efficient loading of a ^210^Fr MOT also by means of local charge transfer induced by contact with a charged dielectric probe (Fig. 1(d)). This effect might provide a new insight into the desorption processes from organic coatings. Results are shown in Fig. 4.

In this configuration, a small plastic rod (length 150 mm, diameter 5 mm) is weakly electrified by triboelectric effect. Once the ^210^Fr MOT population has reached its equilibrium (around 130 atoms in Fig. 4), the charged probe is put in contact with the Pyrex cell wall, whose inner surface is coated with PDMS. Immediately, the number of trapped atoms starts increasing and reaches a maximum of around 1.1 × 10^3^ atoms in 50 s, with a relative increase of 600%, about 3 times larger than in the previous cases of pulsed LIAD. In this case, following the same reasoning as before, the maximum number of ^210^Fr atoms in the vapour phase can be crudely estimated as ${N}_{charge}^{(210)}\approx 2\times {10}^{5}$ atoms.

Thereupon, the MOT population decreases below the initial value, with the same behaviour previously described. This observation further supports the interpretation of the system dynamics after desorption as the interplay between - in this case - adsorbed ions in the coating, vapour and MOT, which is therefore independent from the desorbing mechanism.

In the inset of Fig. 4, the relative variation of the MOT population *N*_*MOT*_(*t*) is plotted as a function of time, with a second application of the plastic rod at t = 161 s:

1 $${\rm{\Delta }}=\frac{{N}_{MOT}(t)-{N}_{MOT}(t=0)}{{N}_{MOT}(t=0)}.$$

Exponential fits of the experimental curves for the two applications of the rod are in qualitative agreement, thus confirming that the two increases of the MOT population are produced by similar mechanisms. The second peak, whose maximum is roughly half of the first one, is explained by the progressive depletion of the cell coating. This effect is widely observed in presence of stimulated desorption^24^ and it is ascribed to the finite density of adsorbed particles in the coating at equilibrium. Incidentally, this further confirms the origin of the additional MOT population, as produced by desorbed Fr previously stored in the PDMS.

We note here that the direct desorption of Fr neutralised by charges and subsequent partial depletion of the coating shown in Fig. 4 is supported - although not conclusively - also by the relevant decrease in the Geiger counts observed right after the application of the rod.

It is important to underline that the desorption is observed only when a *direct* contact of the charged dielectric rod with the cell is established. This fact supports the existence of a flow of charges from the plastic probe to the coating as the leading cause of the enhanced desorption, rather than effects due to a change in the electric field. In other words, extra charges produce an efficient desorption of Fr atoms. We have measured with a picoamperometer a rapid transfer of negative charge $q\mathop{ < }\limits_{ \tilde {}}3\times {10}^{-9}{\rm{C}}$ (~10^10^ elementary charges). Further investigations are required on the details of the charge dynamics in the coating. The diffusion of charges is also consistent with the slower dynamics observed here (loading rate of 22 atoms s^−1^) with respect to the pulsed LIAD case, where the maximum MOT population is achieved in about 5 s (60 atoms s^−1^).

In this case, atomic desorption is therefore explained with an exchange of charges within the complex adsorbed ion-coating. Adsorbed Fr^+^ are bound to locally charged strains of the polymer’s molecular chains. The free charges supplied by the plastic rod diffuse and modify the local interaction and arrangement of the polymer on the Pyrex substrate, thus releasing Fr atoms in the vapour phase. After charge transfer and neutralisation, the energy scale of the adatom-coating interaction at the surface is typically a fraction of eV^25^, therefore accessible to charged-induced re-arrangements of coating as those described here. In other words, whereas LIAD acts on the atomic mobility, the charge transfer mechanism produces neutralisation of Fr and desorption by locally and reversibly altering the adsorbing coating. A similar microscopic mechanism has been proposed also for explaining light-induced desorption of Na in the moon atmosphere^26, 27^.

We note that the results shown in Fig. 4 cannot be interpreted in direct analogy with the case of high electric fields and non-direct contact desorption^28^. In that case, the dynamics appears to be much faster and limited to the application/deactivation of the intense field. Finally, it is noteworthy that - in our case - repeated applications of the charged rod produce repeated increase of the MOT population (see inset of Fig. 4), although with different yield if the initial density of adsorbed Fr^+^ has not been restored, as in this case. This confirms that the coating average properties are unaffected. Consequently, the process is completely reversible and, provided that enough time is given to the system, repeatable: eventually, equilibrium conditions are restored and no permanent modifications of the system are observed.

## Discussion

We have demonstrated the application of Light Induced Atom Desorption from a PDMS coated Pyrex surface to the loading of a MOT of radioactive species. ^209^Fr and ^210^Fr MOTs were loaded by means of pulsed photodesorption from the organic coating produced by a broadband visible light pulse. A fast and efficient response was observed, with about 200% increase in the MOT populations. The process and its dynamics are independent from the isotope in use.

We have also demonstrated MOT loading via atomic desorption induced by charge transfer to the PDMS coating. The enhancement in the loading of ^210^Fr is about 600%. The desorption process reported here, other than previous observations involving large electric fields, relies on a charge transfer from a dielectric probe electrified by triboelectric effect. This phenomenon provides novel insight in the adsorbed particle-coating interaction and in the mechanisms of desorption, with potential impact also in the case of LIAD.

Finally, the results presented here are obtained with a neutraliser at room temperature. Therefore, they prove the feasibility of an alternative path for high-efficiency loading of radioactive and stable MOTs based on controlled desorption from organic coatings. This has potential for a dramatic reduction of power consumption and heat dissipation, two aspects of primary importance in view of applications and miniaturisation of the cold atoms technology.

## Methods

### Francium Isotopes Production and Transport

The experiments are performed at the WADE collaboration’s laser cooling facility at INFN-LNL^10^. Fr is produced by a nuclear fusion-evaporation reaction in a thick Au target, bombarded by ^18^O^6+^ accelerated by the INFN-LNL TANDEM-XTU. Energies between 95 and 115 MeV are reached. The ^197^Au target, contained in a separate scattering chamber because of radioactive hazard, is kept at 1200 K and +3 kV. Fr^+^ ions are thus extracted from the target by electrostatic acceleration. A dedicated electrostatic transport line routes, mass-selects and focuses the desired isotope to the experimental chamber.

### Neutralisation and Magneto-Optical Trap

In the experimental chamber, Fr^+^ ions are neutralised by a 25 *μ* m thick Yttrium foil (“neutraliser”). The Y foil acts as a thermal source of Fr atoms, which are then released in the vapour phase. The MOT cooling light is generated by a Ti:sapph laser, pumped by a CW 20 W Ar^+^ laser. The Ti:sapph source is tuned on the D_2_ line of Fr, 7 S_1/2_ (F = 13/2) → 7 P_3/2_ (F’ = 15/2), at 718 nm. A free running diode laser tuned on the D_1_ line, 7 S_1/2_ (F = 11/2) → 7 P_1/2_ (F’ = 13/2), at 817 nm, acts as a repumper. Trapped atoms are detected by measuring their fluorescence with a cooled calibrated CCD camera, with a final sensitivity of 1 fW, corresponding to 10 atoms^10, 13^.

### MOT Chamber

The MOT chamber is a 150 mm diameter Pyrex sphere, equipped with optical windows for the laser beams. The inner surface is coated by PDMS, in order to minimise atomic losses and to ensure elastic atom-wall collisions. The chamber is continuously evacuated down to <10^−9^ mbar. Remarkably, the probability for an atom to be captured by the PDMS coating with a low energy bond (10^−1^ eV) is nonzero. Depending on the specific coating in use and the quality of the coating procedure, one atom-wall collision out of 10^4^ to 10^6^ leads to a loss in the vapour phase. In this way, the organic polymer becomes a reservoir of loosely bound atoms, at the expenses of the population available for the MOT. Therefore, controlled desorption of adsorbed atoms has the potential to enhance the trapping efficiency.

### Coating Procedure

The Pyrex cell inner surface is carefully cleaned with a solution of methanol (45% in volume), ethanol (45% in volume) and KOH (10% in volume). The cell is rinsed three times with deionised water and evacuated down to 10^−6^ mbar by means of a turbomolecular pump. A preliminary baking up to about 150 °C for a few hours enhances the evaporation rate. Once the vacuum level is stable, the cell is slowly brought to room temperature and pressure. A solution of ether and PDMS (5% in volume) is prepared and then poured into the Pyrex cell. The chamber is carefully spun at about 1 Hz in order to completely wet the inner surface and deposit a uniform layer of organic polymer. The excess solution is removed and the cell is again evacuated by means of a turbomolecular pump. A second baking procedure, not exceeding 100 °C in order to prevent damages to the coating, is performed. After about 6 hours at maximum temperature and pumping rate, the cell is installed.

After installation, the cell inner surface is exposed to a stable alkali vapour (K in the present case) at 10^−7^ mbar for several tens of hours, in order to reduce Fr adsorption. This process in fact ensures passivation of the coating surface and therefore its maximum efficiency. The passivation procedure is monitored by measuring the trapping efficiency with an ^85^Rb MOT, used as an off-line test species for the apparatus^10^. The procedure is repeated, until saturation of the MOT population is observed, thus indicating that the passivation is complete and the PDMS coating has reached its maximum efficiency.
